# Fractional Diffusion Based Modelling and Prediction of Human Brain Response to External Stimuli

**DOI:** 10.1155/2015/148534

**Published:** 2015-05-18

**Authors:** Hamidreza Namazi, Vladimir V. Kulish

**Affiliations:** ^1^Department of Mechanical Engineering, Faculty of Engineering, Universiti Malaysia Sarawak, 94300 Kuching, Sarawak, Malaysia; ^2^School of Mechanical and Aerospace Engineering, Nanyang Technological University, Singapore 639798

## Abstract

Human brain response is the result of the overall ability of the brain in analyzing different internal and external stimuli and thus making the proper decisions. During the last decades scientists have discovered more about this phenomenon and proposed some models based on computational, biological, or neuropsychological methods. Despite some advances in studies related to this area of the brain research, there were fewer efforts which have been done on the mathematical modeling of the human brain response to external stimuli. This research is devoted to the modeling and prediction of the human EEG signal, as an alert state of overall human brain activity monitoring, upon receiving external stimuli, based on fractional diffusion equations. The results of this modeling show very good agreement with the real human EEG signal and thus this model can be used for many types of applications such as prediction of seizure onset in patient with epilepsy.

## 1. Introduction

Brain as the most complex organ in the human body controls all bodies' actions/reactions by receiving different stimuli through the nervous system. Any stimulus stronger than the threshold stimulus is translated by the number of sensory neurons generating information about the stimulus and the frequency of the action potentials. After the action potential has been generated, it travels through the neural network to the brain. In various sections of the network and the brain, integration of the signals takes place. Different areas of the brain respond depending on the kind and location of stimuli. The brain sends out signals which generate the response mechanism.

During many years, numerous studies related to the brain response to external stimuli have been reported by scientists. Some researchers studied the brain response to different kinds of stimuli without proposing any model. In case of visual stimuli, we can mention the work done by Kaneoke et al. in analyzing the effect of the visual stimulus size on the human brain response using magnetoencephalography (MEG) [1]; see also [2, 3]. Other groups of researchers investigated the effect of auditory stimuli on the brain response. For instance, Will and Berg studied and compared the brain responses to periodic stimulations, silence, and random noise using electroencephalography (EEG) [4]; see also [5, 6]. Olfactory stimuli also were the main focus of some researchers. Sutani et al. investigated the brain response to pleasant and unpleasant olfactory stimuli using MEG signals. They found out that the MEG signals have recorded from frontal/prefrontal cortical areas of the brain has some differences in case of pleasant versus unpleasant stimuli [7]; see also [8, 9]. Different works have been reported on the investigation of the brain response to other kinds of stimulus such as emotional stimuli [10, 11] and pain stimuli [12, 13].

On the other hand, some scientists proposed some models of the human brain activity. On the microscopic level, the work done by Freeman in the modeling of the EEG arising from the olfactory bulb of animals during the perception of odors is noteworthy. He developed a set of nonlinear equations for this response which generates EEG like pattern [14, 15]. In another work Seetharaman et al. proposed a mathematical model for generation and propagation of action potential in a node of Ranvier and they called it as the phase-lagging model of single action potential [16]. When the microscopic models are extended to a macrolevel, then different methods are employed. Many of these models assume the cortical region to be a continuum. Liley et al. developed a set of nonlinear continuum field equations which described the macroscopic dynamics of neural activity in the cortical region [17]. These equations were used by Steyn-Ross et al. who introduced noise terms into them to give a set of stochastic partial differential equations (SPDEs). They also converted the equations governed by Liley et al. into linearized ODEs. This model could predict the substantial increase in low frequency power at the critical points of induction and emergence. They later used this model to study the electrical activity of an anaesthetized cortex [18–21]. Kramer et al. started with the equations given by Steyn-Ross and coworkers and neglected the spatial variation and the stochastic input. They believed that this gave rise to a set of ordinary differential equations (ODEs) for the modeling of the cortical activity. They showed that the results obtained from the SPDE model agree with clinical data in an approximate way [22], but they also stated that the spatial sampling of the cortex was poor because of inherent shortcomings in the equipment used. Kulish and Chan have suggested a novel method for the modeling of the brain response using fundamental laws of nature like energy conservation and the least action principle. The model equation obtained has been solved and the results show a good agreement with real EEGs [23].

Despite rapid advances in the studies related to the analysis of the human brain response, there has been less progress in the mathematical modeling of the human brain activity due to external stimuli. Yet, it seems that the contemporary level of developments in physics and mathematics makes establishing quantitative correlations between external physical stimuli and the brain responses to those stimuli.

This paper attempts to introduce a new mathematical model of the human brain response to external stimuli based on the fractional diffusion equation. At first we talk about the macroscopic level of brain organization and EEG signal as an alert state of human behavior monitoring. Then, by introducing fractional diffusion equations and considering the EEG signal as a fractal time series we model the EEG signal using mathematical equations. This model is then solved and after discussing different parameters in the model we provide some results and discuss about the model's solution in details. Some concluding remarks are provided at the end.

## 2. Macroscopic Level of Brain Organization and the EEG Signal

In order to study the human neural activity, one can consider different levels of brain organization at different scales in time and space from a single neuron (microscopic level) to the whole brain organization (macroscopic level). In fact, the whole brain activity cannot be observed by measuring the activity of just a single neuron as far it is informative as it contributes to study the entire population of which it is a member.

In this research, we focus on the macroscopic level of brain organization. Macroscopic level of brain organization refers to the level of neural assemblies' population in which each neural assembly interacts with other neural assemblies in close and distant cortical areas, exhibits spatial-temporal behaviour, and paints the human behaviour [24]. The functional behaviour of the brain is encoded in these spatial-temporal structures and can be extracted from the macroscopic quantities dynamics observed by EEG signal mostly [25].

**Figure 1:**
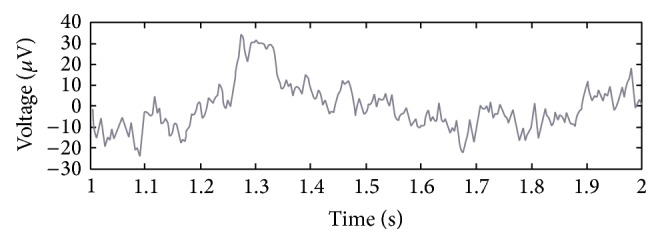
EEG signal of a subject in response to an external stimulus.

During many years scientists have studied the human behavior by recording and analysis of the EEG signals from different areas of the brain. The EEG signal is the composition of different frequency bands (oscillatory activities-Alpha, Beta,…) which are structured coordinately (spatially, temporally). In fact, this signal has different characteristics that can be used in order to study the human brain response to external stimuli in our research. For instance, Figure 1 shows the EEG signal of a subject who received an external stimulus at *t* = 1 s. In this figure the brain response to external stimulus can be seen as a sudden upward deflection at about *t* = 1.25 s after the application of stimulus at *t* = 1 s.

## 3. Fractional Diffusion Equation

Here, in order to develop our model we start with a simple equation:

(1) $$\begin{array}{c} \frac{\partial F}{\partial t}=D\nabla^{2}F, \end{array}$$

where

(2) $$\begin{array}{c} \nabla^{2}F=\frac{\partial^{2}F}{\partial x^{2}}. \end{array}$$

Equation (1) is the well-known diffusion equation where the coefficient *D* is the diffusivity of the medium for the property *F*. In fact, this equation arises in many descriptions of biological and physical phenomena, including Brownian motion [26], gradient driven chemical diffusion (with Fick's law), and heat transfer (heat diffusion with Fourier's Law). It is noteworthy that (1) is written in the Euclidean space.

On the other hand, the diffusion process can also been studied for fractals in fractal space. Fractals (such as random walk) may be defined as self-similar geometric objects whose scaling exponent (dimension) satisfies the Szpilrajn inequality:

(3) $$\begin{array}{c} ℵ\geq D_{T}, \end{array}$$

where *ℵ* is the scaling exponent (dimension) of the object and *D*_*T*_ is its topological dimension, that is, Euclidean dimension of units from which the fractal object is built. In fact, Fractal and Euclidean geometries are conjugate approaches to the geometry of natural forms. Fractal geometry builds complex objects by applying simple processes to complex building blocks; Euclidean geometry uses simpler building blocks but frequently requires complex building processes.

Considering the diffusion process in the case of fractals, (1) is changed to (4) which is called the fractional diffusion equation [27]:

(4) $$\begin{array}{c} \frac{\partial^{2H}F}{\partial t^{2H}}=C^{2\left(2H-1\right)}D^{2\left(1-H\right)}\nabla^{2}F, \end{array}$$

where ∇^2^ *F* = ∂^2^ *F*/∂*x* ^2^.

In (4), *C* is the speed of propagation and *H* is the Hurst exponent with the value within the range 0 ≤ *H* ≤ 1 that brings predictability of signal into account. In fact, the Hurst exponent can be viewed as the probability of the diffusion process being persistent in a certain given direction. Note that the case *H* = 1/2, which corresponds to a nonfractal diffusion process, leads to the well-known classical equation. Observe also that if *H* = 0, (4) degenerates into the Poisson equation, ∂^2^ *F*/∂*t* ^2^ = *C* ^−2^ *D* ^2^∇^2^ *F*; that is, there is no preferred direction of random walk in this case, while the case *H* = 1 leads to the wave equation, ∂^2^ *F*/∂*t* ^2^ = *C* ^2^∇^2^ *F*.

It is now necessary to make a very important remark. It is possible to consider a Brownian motion type process as a process which takes place in an Euclidean space (see the right hand side of (4)), considering the temporal dimension, *t*, of the process as fractal time which, for the same diffusion coefficient, *D*, either slows down (in case of *H* < 1/2) or speeds up (in case of *H* > 1/2) the process in question. This can be described with the generalized diffusion equation (4) in which the time coordinate appears as a fractal quantity. The generalized diffusion equation is a fractional PDE of order 2*H* with respect to time.

Taking all these conjectures into account, (4) is a generalized form for describing the diffusion process when the time becomes fractal.

## 4. Fractional Diffusion Model of EEG Signal

In this section, it is aimed to show that EEG signal as a fractal time series (but not stochastic), which represents a transient record of a random walk process, can be modeled by the solution of the fractional diffusion equation:

(5) $$\begin{array}{c} \frac{\partial^{2H}V}{\partial t^{2H}}=C^{2\left(2H-1\right)}{D_{\text{eff}}}^{2\left(1-H\right)}\frac{\partial^{2}V}{\partial \eta^{2}}, \end{array}$$

where the term *V*, which stands for the brain response, is the voltage fluctuations resulting from ionic current flows within the neurons of the brain. The impulse propagation speed within the neural network, which is a finite quantity, is represented by *C*. The term *D*_eff_, the effective diffusion coefficient, as the property of neural tissue, is related to neuron's resistance to the electrical impulse as it travels over the nerve. The term *H*, as a time variable parameter, corresponds to the Hurst exponent that brings the predictability of signal into account.

**Figure 2:**
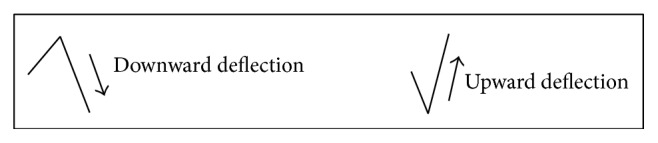
Upward and downward deflection in the signal from one point to the next point.

The direction of deflection at each moment in the signal can be studied by computing the Hurst exponent (Figure 2). The Hurst exponent is an indicator of the long term memory of the process generating the signal and thus it is the measure of the predictability of the signal.

As it was mentioned before the Hurst exponent can have any value between 0 and 1, where the value that it gains in each moment determines the behavior of the next deflection in the signal.

Firstly, if the Hurst exponent has a value between 0 and 0.5, it means that the process is antipersistent; that is, the trend of the value of the process at the next instant will be opposite to the trend in the previous instant. Secondly, a value of *H* between 0.5 and 1 means that the process is persistent; that is, the trend of the value of the process at the next instant will be the same as the trend in the previous instant. Finally, if *H* = 0.5, the process is considered to be truly random (e.g., Brownian motion). It means that there is absolutely no correlation between any values of the process.

One of the interesting points about the fractional diffusion model (5) is that it accounts for a finite time lag (reaction time) between any given disturbance (stimulus) and the brain response to it (human action/reaction) based on the assumption that no instantaneous propagation of information is possible within the brain. This effect is considered during the derivation of this model, but it is substituted by *C*, *D* according to the equation:

(6) $$\begin{array}{c} \tau =\frac{D_{\text{eff}}}{C^{2}}. \end{array}$$

Equation (6) is the formula for relaxation time in hyperbolic reaction diffusion equations [28].

In this research we consider the brain in an informational space where *η* is the spatial dimension of this space. In fact, each two neurons in informational space, which have informational interlink, make an informational channel between themselves and the length of this channel is called the informational distance *η* where

(7) $$\begin{array}{c} 0\leq \eta <\infty . \end{array}$$

It is noteworthy that informational distance is not same as the concept of spatial distance. Two neurons, which are close together, may not exchange information which means *η* ~ *∞*. On the other hand, two neurons which are far apart may be closely interlinked to each other and exchange a lot of information which makes *η* very small. Thus, *η* has value in the range 0 ≤ *η* ≤ *∞* [29].

The exchange of information between two neurons in the brain happens when there is a potential difference along the channel. In fact, the potential difference between two neurons causes an information flux and the exchange of information from the neuron with higher informational potential value to the one with the lower value continues till the gradient becomes zero.

The fractional diffusion equation is valid for the time scale:

(8) $$\begin{array}{c} t\geq 0. \end{array}$$

Also, regarding (5),

(9) $$\begin{array}{c} V\left(\eta ,0\right)=V_{0} \end{array}$$

is the initial condition at *t* = 0. Also, the solution of the model is required to be bounded as *η* → *∞*. Otherwise, the conservation of energy principle would be violated. In other words,

(10) $$\begin{array}{c} \underset{\eta \to +\infty }{\lim}V\left(\eta ,t\right)=\text{const}<\infty \end{array}$$

is the boundary condition in the case of infinite domain.

In order to solve the above fractional diffusion model the method proposed by Oldham and Spanier is employed here [30].

Upon introducing the excess value $\hat{V}\left(\eta ,t\right)=V\left(\eta ,t\right)-V_{0}$, so the initial condition imposed on $\hat{V}$ is $\hat{V}\left(\eta ,0\right)=0$.

In order to solve (5) we apply the Laplace transform with respect to time, *t*; then we have

(11) $$\begin{array}{c} C^{2\left(2H-1\right)}{D_{\text{eff}}}^{2\left(1-H\right)}\frac{\partial^{2}Y}{\partial \eta^{2}}-s^{2H}Y=0, \end{array}$$

where *s* is the Laplace transform variable and *Y* denotes the Laplace transform of the excess value $\hat{V}$.

Equation (11) is a second-order ordinary differential equation (ODE), where the general solution is calculated as

(12) Yη;s=A1se−ηsH/C2H−1Deff1−H +A2seηsH/[C2H−1Deff1−H],

where *A*_1_(*s*) and *A*_2_(*s*) are two arbitrary functions. However, since the solution is to be bounded for all *η*, the second arbitrary function, *A*_2_(*s*), must be identically zero, so the solution (12) is changed to

(13) $$\begin{array}{c} Y\left(\eta ;s\right)=A_{1}\left(s\right)e^{-\eta s^{H}/{[C}^{\left(2H-1\right)}{D_{\text{eff}}}^{\left(1-H\right)}]}. \end{array}$$

Upon differentiating (13) with respect to *η*,

(14) $$\begin{array}{c} \frac{dY\left(\eta ;s\right)}{d\eta }=-\frac{A_{1}\left(s\right)s^{H}e^{-\eta s^{H}/{[C}^{\left(2H-1\right)}{D_{\text{eff}}}^{\left(1-H\right)}]}}{\left[C^{\left(2H-1\right)}{D_{\text{eff}}}^{\left(1-H\right)}\right]}. \end{array}$$

After comparing (13) and (14), *A*_1_(*s*) can be eliminated; then it can be written as

(15) $$\begin{array}{c} Y\left(\eta ;s\right)=-s^{-H}C^{\left(2H-1\right)}{D_{\text{eff}}}^{\left(1-H\right)}\frac{dY\left(\eta ;s\right)}{d\eta }. \end{array}$$

By taking the inverse Laplace transform of (15) and restoring the original variables, then we have

(16) $$\begin{array}{c} V\left(\eta ,t\right)=V_{0}-C^{\left(2H-1\right)}{D_{\text{eff}}}^{\left(1-H\right)}\frac{\partial^{-H}}{\partial t^{-H}}\left(\frac{\partial V}{\partial \eta }\right) \end{array}$$

which is written in terms of fractional derivative of order −*H* with respect to *t*.

Using the definition of fractional derivative [30], namely,

(17) ∂αf∂tα=1Γ−α∫0tfξdξt−ξα+1, Reα<0,

where Γ(*α*) is the Gamma function, and noticing the Fick's law,

(18) $$\begin{array}{c} -\frac{\partial V}{\partial \eta }=\frac{\varphi }{D_{\text{eff}}}, \end{array}$$

where *φ* represents the flux, and (16) can be written as

(19) $$\begin{array}{c} V\left(\eta ,t\right)=V_{0}+C^{\left(2H-1\right)}{D_{\text{eff}}}^{\left(-H\right)}\frac{1}{\Gamma \left(H\right)}\overset{t}{\underset{0}{\int }}\frac{\varphi \left(\eta ,\xi \right)d\xi }{{\left(t-\xi \right)}^{1-H}}. \end{array}$$

Since the function *φ*(*η*, *t*) represents the flux it can be equated with the external influence acting on the system (external stimulus).

Equation (19) provides the relationship between the nonequilibrium value, *V*(*η*, *t*), and the external influence acting on the system, *φ*(*η*, *ξ*). This equation is valid for every location within the domain (including the boundary) at every moment.

Since *H* is a nonnegative parameter, it follows from (19) that the value of *V*, on the average, increases with the time according to the power law as *t* ^*H*^, provided of course that the fluctuations are small in comparison with the averaged influence. Note that, for *H* = 1/2, Γ(1/2) = *π* ^1/2^ and (19) yields the well-known diffusion (random walk) growth given by *t* ^1/2^.

An external influence can be modeled by a Gaussian pulse; that is,

(20) $$\begin{array}{c} \varphi \left(x,t\right)=\varphi_{0}\left(x,t\right)\exp{\left[-\frac{{\left(t-t^{*}\right)}^{2}}{\sigma^{2}}\right]}^{}, \end{array}$$

where *t* ^∗^ denotes the moment, at which the Gaussian pulse reaches its maximal value *φ*_0_(*x*, *t*), whereas *σ* is the standard deviation of the Gaussian pulse.

In case of different types of stimuli, depending on the size and duration, the parameters in (20) will have different values. In the case of many concurrently external stimuli, a series of Gaussian pulses is considered.

It is noteworthy that (20) is not a compulsory form of external stimulus where other formulas might be used, provided that they meet the principle demands discussed in this research. This mathematical form is chosen because its capability was examined in the modeling of generation of action potential in a neuron [16].

In the next section we provide a formulation for computing the effective diffusion coefficient at each moment that can be used in (19) in order to compute the brain response to external stimuli.

## 5. A Phase-Lagging Diffusion Based Model of the Diffusion Coefficient

In order to analyze the behavior of fractal time series, the value and the direction of each fluctuation in the signal should be analyzed. The direction of each fluctuation can be found by computing the value of the Hurst exponent at the previous point of the signal.

In order to know about the value of the signal, we should know about different parameters which are appeared in the fractional diffusion model. By looking at (19) and replacing the value of *φ* = −*D*_eff_(∂*V*/∂*η*), we have

(21) $$\begin{array}{c} V\left(\eta ,t\right)=V_{0}-C^{\left(2H-1\right)}{D_{\text{eff}}}^{\left(1-H\right)}\frac{1}{\Gamma \left(H\right)}\overset{t}{\underset{0}{\int }}\frac{\partial V}{\partial \eta }\frac{d\xi }{{\left(t-\xi \right)}^{1-H}}. \end{array}$$

The value of the Hurst exponent which is used in (21) can be computed using MATLAB based on Rescaled Range Analysis method [31, 32]. Then, in order to compute the value of the signal in (21) two parameters *C* and *D* should be known. In order to do this, first, a relationship between *D* and *H* can be made, and then by using the formula for relaxation time, (6), *C* can be replaced by *D* and *τ*.

In order to make a relation between *D*_eff_ and *H*, the phase-lagging model of action potential can be used [16]:

(22) $$\begin{array}{c} V\left(x,t\right)=V_{0}-{\left(\frac{D}{\tau }\right)}^{1/2}\overset{t}{\underset{0}{\int }}\frac{\partial V}{\partial x}T_{0}\left(\frac{t-\xi }{2\tau }\right)\exp\left(-\frac{t-\xi }{2\tau }\right)d\xi . \end{array}$$

In (22) the diffusivity term *D* is related to the resistance of the neuron to the electrical impulse. *D* is the property of the neural tissue and will dampen the impulse as it travels over the nerve. The term *τ* is the reaction time and *𝔗*_0_(*z*) is the zero-order modified Bessel function.

Considering the phase-lagging model of action potential in the whole brain scale then, this model and the fractional diffusion model explain the same phenomenon. Thus, the mathematical equations that belong to these two models can be equal.

By writing this equality,

(23) V0−Dτ∫0t∂V∂ηT0t−ξ2τexp⁡−t−ξ2τdξ =V0−C2H−1Deff1−H1ΓH∫0t∂V∂ηdξt−ξ1−H,

where *D* is the diffusion coefficient in the phase lagging model of action potential and *D*_eff_ is the diffusion coefficient in the fractional diffusion model.

Considering *C* = (*D*_eff_/*τ*)^1/2^ and removing *V*_0_ from both sides of the equation,

(24) Dτ∫0t∂V∂ηT0t−ξ2τexp⁡−t−ξ2τdξ  −τ1/2−HDeff1ΓH∫0t∂V∂ηdξt−ξ1−H=0.

Then, we can write

(25) ∫0tDτ∂V∂ηT0t−ξ2τexp⁡−t−ξ2τ   Dτ∂V∂ηT0t−ξ2τexp−t−ξ2τ−τ1/2−HDeff1ΓH∂V∂η1t−ξ1−Hdξ=0.

Because (25) is valid for all values of *t* > 0 then we can remove the integral:

(26) Dτ∂V∂ηT0t2τexp⁡−t2τ −τ1/2−HDeff1ΓH∂V∂η1t1−H=0.

Thus,

(27) Dτ∂V∂ηT0t2τexp⁡−t2τ =τ1/2−HDeff1ΓH∂V∂η1t1−H.

Diving both sides of (27) by ∂*V*/∂*η* ≠ 0 and introducing a new variable *z* = *t*/2*τ* we have

(28) $$\begin{array}{c} \frac{D_{\text{eff}}}{D}={\left[2^{1-H}\Gamma \left(H\right)z^{1-H}T_{0}\left(z\right)\exp\left(-z\right)\right]}^{2}. \end{array}$$

**Figure 3:**
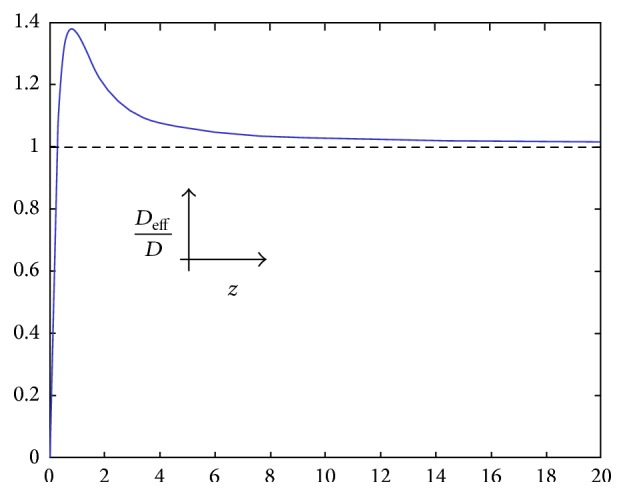
Dependence of the dimensionless diffusivity, *D*_eff_/*D*, on the dimensionless temporal variable, *z*, for *H* = 0.5.

**Figure 4:**
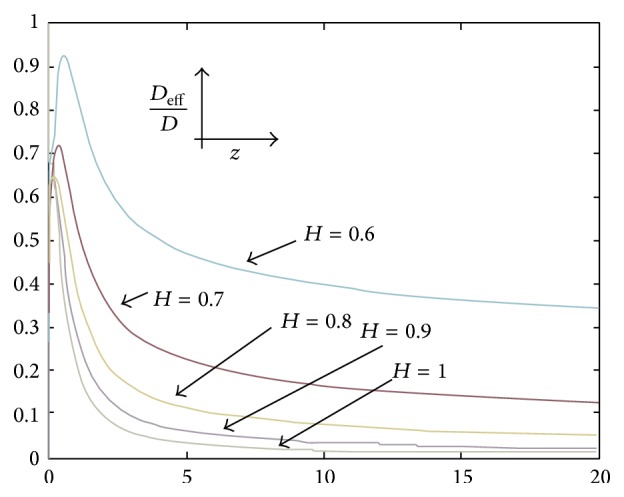
Dependence of the dimensionless diffusivity, *D*_eff_/*D*, on the dimensionless temporal variable, *z*, in case of 0.5 < *H* ≤ 1.

Considering a constant value for *D*, in order to study the variation of *D*_eff_/*D* versus *z*, Figures 3 and 4 are provided. It is noteworthy in order to analyze the value of the signal that it is only needed to concentrate on the calculations of *D*_eff_ and, accordingly, *V* in 0 ≤ *H* ≤ 0.5 or 0.5 ≤ *H* ≤ 1 because as it is known, having the probability of *H* on one side yields the probability of *H*′ = 1 − *H* in the reverse side, but with the same value of fluctuation. In this research the span 0.5 ≤ *H* ≤ 1 is taken as the reference. So, for instance, in order to compute the value of the signal in a point with *H* = 0.2 it is needed just to compute the value of the signal with *H* = 0.8.

As it can be seen in Figures 3 and 4, first the value of dimensionless diffusivity increases but after that as time increases (*z* → *∞*), dimensionless diffusivity tends a constant value. In order to describe this behavior, (28) can be analyzed when *z* → *∞*:

(29) $$\begin{array}{c} \underset{z\to \infty }{\lim}\frac{D_{\text{eff}}}{D}={\underset{z\to \infty }{\lim}\left[2^{1-H}\Gamma \left(H\right)z^{1-H}T_{0}\left(z\right)\exp\left(-z\right)\right]}^{2}. \end{array}$$

$\underset{}{\lim_{z\to \infty }}{𝔗}_{0}\left(z\right)$ is computed as

(30) $$\begin{array}{c} \underset{z\to \infty }{\lim}T_{0}\left(z\right)=\frac{\exp\left(z\right)}{\sqrt{2\pi z}}=\exp\left(z\right)2^{-1/2}\pi^{-1/2}z^{-1/2}. \end{array}$$

By substituting (30) into (29),

(31) $$\begin{array}{c} \underset{z\to \infty }{\lim}\frac{D_{\text{eff}}}{D}=\underset{z\to \infty }{\lim}\left[\frac{2^{1-2H}\Gamma^{2}\left(H\right)}{\pi }z^{1-2H}\right]. \end{array}$$

Considering (31) in case of *H* = 0.5

(32) $$\begin{array}{c} \underset{z\to \infty }{\lim}\frac{D_{\text{eff}}}{D}=\underset{z\to \infty }{\lim}\left[1\right]=1 \end{array}$$

which is the same as the trends observed in Figure 3.

By considering (31) in case of 0.5 < *H* ≤ 1 we have

(33) $$\begin{array}{c} \underset{z\to \infty }{\lim}\left[\frac{2^{1-2H}\Gamma^{2}\left(H\right)}{\pi }z^{1-2H}\right]=\underset{z\to \infty }{\lim}\left[X\cdot \frac{1}{z^{2H-1}}\right]. \end{array}$$

Thus,

(34) $$\begin{array}{c} \underset{z\to \infty }{\lim}\frac{D_{\text{eff}}}{D}=0 \end{array}$$

which is the same as the trend observed in Figure 4 for values of *H* where 0.5 < *H* ≤ 1. So, in both cases (*H* = 0.5 and 0.5 < *H* ≤ 1) the dimensionless diffusivity tends to a constant value as time increase.

So, in cases of Figures 3 and 4, before the maximum point of graph, as time goes on, the value of the dimensionless diffusivity increases, but after passing the maximum point, the dimensionless diffusivity shows the opposite behavior and, after some time, the dimensionless diffusivity tends to a constant value which is 0 and 1 in the cases of 0.5 < *H* ≤ 1 and *H* = 0.5, respectively.

As *D*_eff_ has been considered as a time dependent parameter and *D* as a constant, thus from previous discussion it can be concluded that when human senses a stimulus, the diffusion of this stimulus to human brain increases but after some time, the diffusion of the stimulus decreases. In fact, these results validate and show the importance of (28) for computing the value of *D*_eff_.

Thus, by computing the value of *D*_eff_ at each time moment and substituting its value in the fractional diffusion model the value of the signal at each time moment can be computed.

## 6. Result and Discussion

In this section using the fractional diffusion model the human brain response to a visual external stimulus is modeled and compared with the real EEG signal.

### 6.1. Subjects

In this research the experiments were carried out on 6 voluntary healthy students (21–24 years old, 3 males and 3 females). Prior to the experiment, each subject was examined and interviewed by a physician to ensure that no neurological deficit, pain condition, or medication affects the EEG. All subjects were right handed. Informed consent was obtained from each subject after the nature of the study was fully explained.

All procedures were approved by the Internal Review Board of the University and the approval for experimentation involving human subjects was issued. It is noteworthy that the identity of all subjects remains confidential.

### 6.2. EEG Recording

The EEG data used in this research were collected using Mindset 24 device, a 24-channel topographic neuromapping instrument, which can measure 24 channels of data with the sampling frequency of 256 Hz.

**Figure 5:**
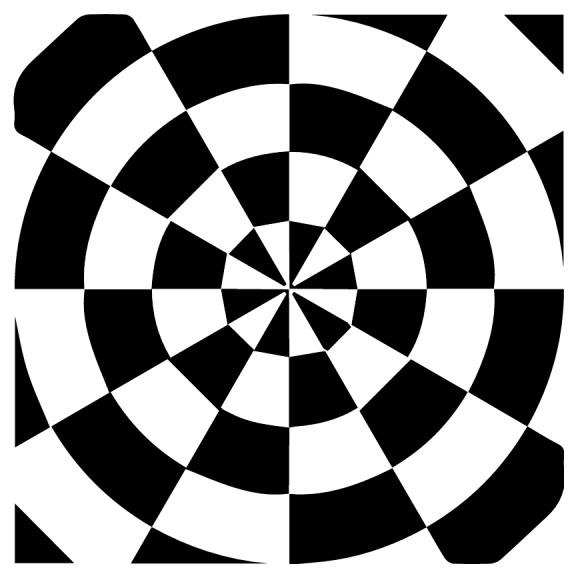
Checkerboard pattern.

In an electrically shielded, acoustically isolated, and dimly illuminated room a visual stimulus applied on subjects. It should be mentioned that it is endeavoured to insulate the subjects from all other external stimuli. This ensures that the response measured in the EEG signals is primarily due to the stimulus applied. In the experiment subjects were watching a checkerboard pattern (see Figure 5) on the monitor of a computer from the distance of 130 cm.

**Figure 6:**
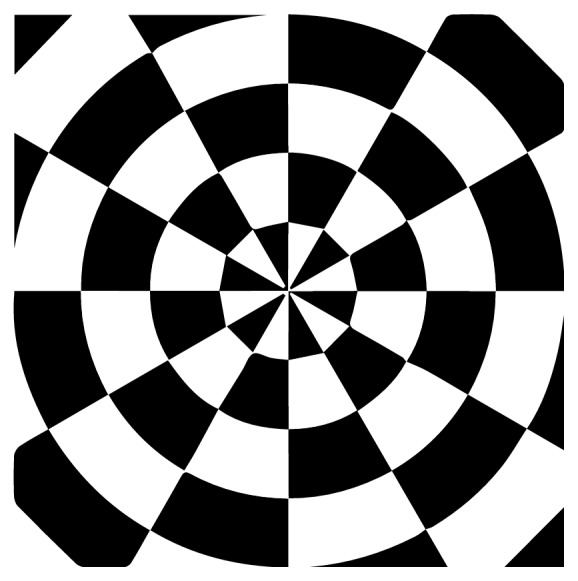
The reversed pattern as the visual stimulus.

The stimulus was the checker reversal. After one second the reversed pattern (see Figure 6) was displayed and then the original pattern was displayed again. Thus, the stimulus was applied at *t* = 1 s. The interstimulus interval of 5 s was chosen in these experiments.

Mindmeld 24 software is used for the collection of data using Mindset 24 machine. The software gives data in the form of  .bin files which can be processed to give text files (.txt) that are required for further processing.

Although EEG data are recorded from 24 electrodes, in this research the analysis is done on the data governed from the left occipital (O1) electrode (near to the location of the visual primary sensory area). This electrode was chosen based on the nearest place to the visual sensory area which shows the strongest response that can be seen in the signal recorded from this electrode compared to other electrodes. The electrode impedance was kept lower than 5 KΩ.

A bipolar electrooculogram (EOG, vertical and horizontal) was recorded for off-line artifact rejection. After bandpass filtering in the range of 0.1–70 Hz, 2 seconds of data (256 data before stimulation and 256 data after stimulation) was saved. It means that there are 256 values of voltage collected every second. Prestimulation is defined as the status before the application of the stimulus. On the other hand, poststimulation is defined as the status after the application of the stimulus. As it was mentioned previously the stimulus was applied at *t* = 1 s.

It is noteworthy that choosing higher sampling rate will result in more pre- and poststimulation data. Having more data will results in more precise computation of Hurst Exponent, diffusion coefficient, and accordingly the signal and its parameters such as response initiation time.

In the first week 40 trials were collected from each subject in one day. The data collections were repeated after a week for each subject in order to examine the reproducibility of the results from experiments. By repeating the experiments in the second week totally 80 trials were collected. After visual inspection of data collected from each subject and rejection of trials with artifacts, 40 trials free of artifacts were selected for future analysis. It is noteworthy mentioning that physician monitored the subjects during all experiments.

### 6.3. Data Analysis

A set of codes was written in MATLAB software in order to compute all required parameters which were discussed before.

As the recorded data were noisy, the EEG signals were filtered using the Wavelet toolbox in MATLAB and then were processed by the methodology discussed in this section.

The value of *V*_0_ can be read from the record of the signal at the moment the stimulus is applied to the subject, *t* = 1 s. The initial value of *H* is computed for 1 second of the recorded data before the application of the stimulus to the subject. In order to compute the Hurst exponent, as it was mentioned previously the Rescaled Range Analysis method is employed, which is widely used by statisticians.

It is required to compute the Hurst exponent value in each moment in order to analyze the generated signal. At the first step, the program computes the Hurst exponent for the recorded EEG signal and the predicted signal and generates two time series in one figure.

In each moment, the program computes *D*_eff_ using (28). Also, as it was mentioned previously that, for all analysis performed here, a single stimulus is considered and thus a single Gaussian pulse is modeled using (20). By substituting the required parameters in (35), the program computes the value of the signal in each moment and then plots the modeled signal in a figure together with the recorded EEG signal (after stimulation):

(35) $$\begin{array}{c} V\left(\eta ,t\right)=V_{0}+\tau^{1/2-H}\frac{1}{\sqrt{D_{\text{eff}}}}\frac{1}{\Gamma \left(H\right)}\overset{t}{\underset{0}{\int }}\frac{\varphi \left(\eta ,\xi \right)d\xi }{{\left(t-\xi \right)}^{1-H}}. \end{array}$$

It is noteworthy that (35) is governed by substituting *C* = (*D*_eff_/*τ*)^1/2^ into (19).

The values of some required parameters are listed in Table 1.

As it was mentioned previously, one second of EEG data was recorded, and using these data as the reference, one second of EEG time series is predicted. After that, the modeled signal is analyzed in terms of the initiation time for the response, the response duration, and the peak to peak voltage. In this research in order to see the response fluctuations clearly, the real or the modelled signals after the poststimulation are averaged in each case. It means that for each subject 40 selected trials are used as the input to the model and, accordingly, the grand average of all modeled signals (40 signals for each subject) is presented. Then, the generated plots for the real or the predicted signals are the result of averaging over all selected trails in each case.

Here it should be mentioned that in the analyses of the EEG signals plots the initiation time for the response and the duration are chosen based on the literature notes which consider the major positive or negative pole. For instance, the fluctuations which have voltage in the range of 5 to 10 *μ*V or −5 to −10 *μ*V are related with the response to the stimulus. Thus, for instance, in order to have a feature of the response duration, the time span between the first peak and the last peak which have the voltage values within one of these two ranges is considered.

**Figure 7:**
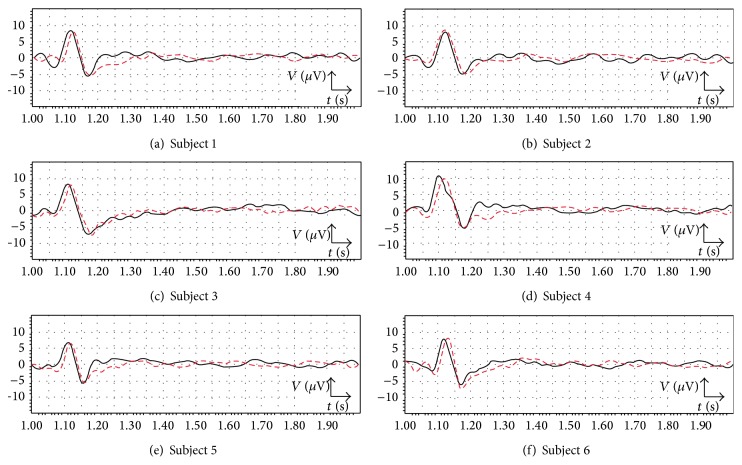
The grand average of the recorded EEG signals (black solid line) and the grand average of the predicted signals (red dashed line) for 1 second after stimulation in the case of the visual stimulus, subject 1 to subject 6.

The grand average of the recorded EEG signals and the grand average of the predicted signals using the fractional diffusion model over all selected trials for 1 second post-stimulation are shown in Figure 7.

As it can be seen for different subjects in Figure 7, the recorded signal (black solid line) and the predicted signal (red dashed line) show the similar behavior. In both cases the brain response to the stimulus starts with a positive peak (*P*) after the application of the stimulus to the subject where its amplitude goes further than 5 *μ*V. This response causes the signal's voltage to fluctuate in a bigger span. Following the positive peak a negative rebound (*N*) can be seen in the plot. In fact, the response to the stimulus terminates at this negative peak, after which the brain goes back to its normal status during rest, without any big deflection in the signal.

As an example, for subject 1 in cases of the real and predicted signals the response to the stimulus starts with a positive peak (*P*) at about 118 ms and about 127 ms, respectively, after the application of the stimulus to the subject. This response causes the signal's voltage to fluctuate in a bigger span. Following the positive peak a negative rebound (*N*) at about *t* = 1.170 s and *t* = 1.174 s can be seen in the plot in cases of the real and predicted signals, respectively. In fact, the response to the stimulus terminates at this point, after which the brain goes back to its normal status during rest, without any big deflection in the signal.

The values of peak to peak voltage, the initiation time for the response (the initial peak of the response), and the response duration (the time difference between the first and the last peaks within the response duration) for the recorded EEG signal and the predicted signal in case of different subjects are provided in Table 2.

**Figure 8:**
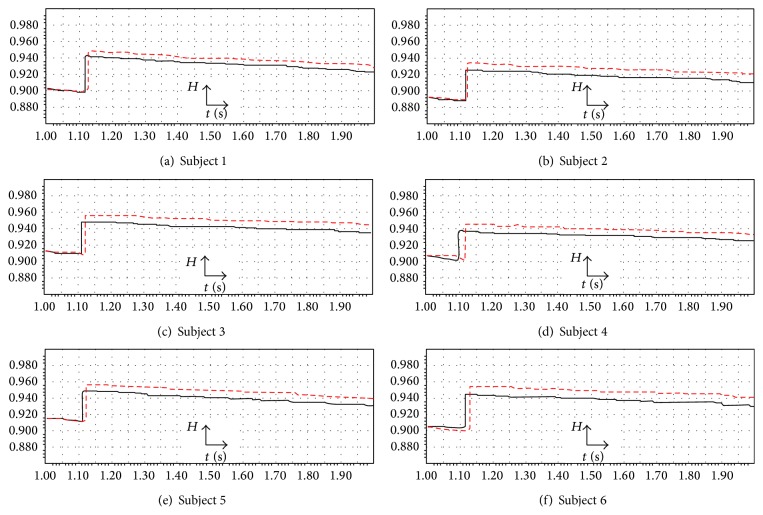
The grand average of the Hurst exponent variations for the recorded EEG signals (black solid line) and the grand average of the Hurst exponent variations for the predicted signals (red dashed line) for 1 second after stimulation in the case of the visual stimulus, subject 1 to subject 6.

As it can be seen in Table 2 for all subjects the predicted initiation time for the response, the response duration, and the peak to peak voltage have very close values with their related values in the recorded EEG signals. Thus, it can be said that the predicted signal resembles the real EEG signal within the response duration in the cases of the initiation time for the response, the peak to peak voltage, and the response duration. Moreover, in order to study the uncertainty and predictability of the model's solution, the Hurst exponent variations for the recorded EEG signals and the predicted signals over all selected trials for 1 second after stimulation are shown in Figure 8.

The high correlation between the values of the real signals and also the predicted signals can be realized by looking at the values of the Hurst exponents. For instance, in the case of subject 1, the value of the Hurst exponent is distributed between 0.900 and 0.943 for the recorded EEG signal (black solid line) and between 0.900 and 0.952 for the predicted signals (red dashed line). Thus, the low uncertainty of the prediction can be confirmed, and it can be said the signal is predicted well, because the Hurst exponent values are not close to *H* = 0.5, which stand for a truly random process. This behavior can be seen in the Hurst exponent plots for all subjects.

Also, as it can be seen in the Hurst exponent plot, the value of the Hurst exponent in the case of the real EEG signal and the predicted signal experiences a sudden upward deflection. For instance, in the case of subject 1, the value of the Hurst exponent is decreasing in the time span of *t* = 1 s to about *t* = 1.118 s and *t* = 1 s to about *t* = 1.127 s, respectively, for real EEG signal and the predicted signal; after that, a sudden upward deflection can be seen, which stands for experiencing the visual stimulus, and again the trend shows the same behavior. The overall decreasing behavior stands for the phenomenon that when a longer time span is considered, the less the human brain “remembers” its initial state. The same behavior can be seen in other Hurst exponent plots.

By analyzing the behaviors which have been seen in Figures 7 and 8 it can be said that on one hand the uncertainty of the prediction was low and on the other hand, the accuracy of the prediction was very good as the predicted signal resembles the real EEG signal.

All the analyses which have been done in this research show that EEG signals can be modeled by the solution of fractional partial differential equations and, thus, the behavior of system modeled by means of such equations can, in principle, not only be predicted but also quantifies.

## 7. Conclusion

In this paper we introduced a new mathematical model which quantifies the human brain response to external stimuli. We developed this model by applying the fractional diffusion equation to human EEG signals. The model generates a multifractal time series which shows a quantitative concurrence with the real EEG signals. Using this model we successfully predicted the EEG signals of different subjects upon receiving a visual stimulus. This model shall be further applied in case of different external stimuli where the results can be verified against the real EEG signal which means the prediction of the human behavior by forecasting the EEG signal. On the other hand this model also can be employed in order to predict different abnormal brain activities, such as epileptic seizure, by at least some seconds before the time of occurrence. If so, a seizure warning and the expected time of this epilepsy occurrence can be generated, leading to the future monitoring of this disease.

## Figures and Tables

**Table 1:** Values of required parameters.

Variable	Value	Units
*D*	6.5 × 10^−4^	m^2^/s
*φ*_0_(*x*, *t*)	1	V · m/s
*t* ^∗^	0.002	s
*σ*	0.001	s

**Table 2:** Comparison between the real and the predicted signals.

Subject	The initiation time for the response (ms)		Response duration (s)		Peak to peak voltage (*μ*V)	
	Real	Predicted	Real	Predicted	Real	Predicted
1	P118	P127	0.052	0.047	13.97	13.07
2	P120	P120	0.054	0.067	12.86	13.75
3	P110	P120	0.058	0.060	15.24	15.24
4	P100	P120	0.078	0.057	16.28	15.50
5	P112	P121	0.043	0.041	12.50	11.81
6	P120	P130	0.050	0.044	13.85	15.50
Average	**113**	**123**	**0.055**	**0.052**	**14.11**	**14.14**
